# Recent advances in understanding and managing epidermolysis bullosa

**DOI:** 10.12688/f1000research.14974.1

**Published:** 2018-07-17

**Authors:** Dimitra Kiritsi, Alexander Nyström

**Affiliations:** 1 Department of Dermatology, Medical Center-University of Freiburg, Faculty of Medicine, 79104 Freiburg, Germany

**Keywords:** skin fragility, collagen VII, laminin-332, gene therapy, TGF-b signaling

## Abstract

Epidermolysis bullosa (EB) is a clinically and genetically heterogeneous skin fragility disorder characterized by trauma-induced skin dissociation and the development of painful wounds. So far, mutations in 20 genes have been described as being associated with more than 30 clinical EB subtypes. The era of whole-exome sequencing has revolutionized EB diagnostics with gene panels being developed in several EB centers and allowing quicker diagnosis and prognostication. With the advances of gene editing, more focus has been placed on gene editing-based therapies for targeted treatment. However, their implementation in daily care will still take time. Thus, a significant focus is currently being placed on achieving a better understanding of the pathogenetic mechanisms of each subtype and using this knowledge for the design of symptom-relief therapies, i.e. treatment options aimed at ameliorating and not curing the disease.

Epidermolysis bullosa (EB) is a clinically and genetically heterogeneous skin fragility disorder characterized by trauma-induced skin dissociation and the development of painful wounds ^1^. Based on the ultrastructural split level, four main EB types exist: EB simplex (EBS) with an intraepidermal split, junctional EB (JEB) with separation within the lamina lucida, dystrophic EB (DEB) with sublamina densa separation, and Kindler syndrome with various split levels. So far, mutations in 20 genes have been described as being associated with more than 30 clinical EB subtypes ^2^. This correlates with the complexity of the disease, since mutations within the same gene may cause more- or less-severe phenotypes of the disease with variable systemic manifestations complementing the skin manifestations. Mutations in the gene encoding collagen VII, for example, may cause only dystrophic nails, but they can also be associated with generalized blistering of the skin, severe scarring after healing of wounds, and numerous associated complications and organ manifestations ^3, 4^. To allow some prognostication, a thorough diagnosis is needed. This requires taking a skin biopsy and performing antibody-mediated staining for the proteins known to be associated with EB—a method called immunofluorescence mapping—or performing electron microscopy analysis. Both of these methods can be offered only by highly specialized EB centers. The era of whole-exome sequencing has revolutionized EB diagnostics with gene panels for EB being established in genetic departments, allowing a rapid and efficient diagnosis for patients with suspected EB ^5, 6^. Unfortunately, the treatment for individuals with EB is still symptomatic with implementation of modern wound care and treatment of the disease-associated complications ^7– 9^.

For years, research on EB has focused on identifying the disease-causing genes, allowing comprehensive classification of the clinical manifestations of each subtype and first approaches for the development of targeted therapies. The accessibility of the skin and the ability to expand epidermal stem cells in culture initially led to a focus on employing epidermal skin grafts for treatment. Major advancements have been made for these approaches. In 2006, an Italian research group was successful in achieving long-term engraftment of epidermal sheets generated from *ex vivo*, gene-corrected, autologous epidermal stem cells from a patient with JEB caused by *LAMB3* mutations ^10^. Safety concerns regarding the use of retroviral vectors put subsequent studies on hold. However, these gene therapy efforts have recently been revived, and two publications from the research group, using the same approach of correction, have repeated the initial success of achieving long-term closure of chronic wounds ^11^ as well as significantly advanced the treatment by successfully replacing 80% of the epidermis of a boy with laminin-332-deficient JEB ^12^. DEB is caused by mutations in the collagen VII gene ( *COL7A1*) and characterized by blistering at the subluminal densa. Transplantation of epidermis generated from *ex vivo* corrected autologous keratinocytes has also shown promise in a clinical trial for the treatment of chronic wounds in DEB. Epidermal stem cells deficient in collagen VII were corrected by transduction of a retroviral vector carrying *COL7A1* as the transgene. Multiple wounds on four patients were treated, and for all patients initial healing of grafted wounds was observed ^13^. However, there were indications that the benefit was less sustained than for laminin-332-deficient JEB ^13, 14^. The reasons for the differences still need to be clarified, but they could involve the basic biology of the proteins, with laminin-332 in skin being an epidermal product only and collagen VII being contributed by both the epidermis and the dermis ^14^. Presently, direct *in vivo* mediated gene correction to the skin using a herpes simplex virus-1 vector is being developed for DEB. It has undergone late-phase pre-clinical studies, and clinical testing is being prepared.

Additional approaches using either allogeneic wild-type bone-marrow-derived stem cells ^15^ or autologous spontaneously corrected epidermal stem cells, e.g. revertant mosaic patches, are possible therapeutic strategies that would not require gene engineering ^16^.

With the advances of gene editing, together with the complex organization, regulation, and steps needed for the functional synthesis of extracellular matrix proteins associated with EB, it is expected that more focus will be placed on gene editing-based therapies for targeted treatment. However, their implementation in daily care will still take time. Thus, a significant focus is currently being placed on achieving a better understanding of the pathogenetic mechanisms of each subtype and using this knowledge for the design of symptom-relief therapies, i.e. treatment options aimed at ameliorating and not curing the disease.

Patients with the most severe forms of DEB present with widespread blistering and the development of chronic, painful wounds. Healing occurs with the development of scars and fibrosis, leading to acral contractures and pseudosyndactyly ^17^. Besides the skin, the mucosa is affected as well, and patients commonly suffer from esophageal stenoses with severe underweight and organ manifestations, including in the heart, liver, and kidney. We and others ^18– 20^ have recently shown that TGF-β signaling activity is a major determinant of disease severity in DEB. DEB manifested differently in a monozygotic twin pair owing to the differential presence in their skin of decorin, a molecule known to trap TGF-β ^20^, making it inaccessible for signaling through its cognate receptors. In addition, elevated levels of FGF have been observed in individuals with recessive dystrophic EB (RDEB) ^21^. This, together with the enhanced TGF-β, has been proposed to result in increased vascular density of RDEB skin ^22^, thus creating a tumor-promoting microenvironment. An even further tumor-susceptible microenvironment is created by the bacterial overload and/or diversity loss of RDEB skin ^23, 24^, which is partially due to an insufficient innate immune response to bacteria ^4^. Increased abundance of flagellated bacteria has been shown to be a mediator of wound-induced carcinogenesis ^25^. Furthermore, studies have also suggested that bacteria are involved not only in the initiation of tumors but also in their conversion to a more aggressive and invasive phenotype ^26^. Based on the elevated TGF-β levels and activity in DEB ^27^, as well as in the DEB mouse model, methods to target this therapeutically were sought. Losartan, an angiotensin II type 1 receptor antagonist, was fed to the DEB mouse model over a period of 7 weeks, and the effects were compared to untreated mice ^18^. A significant reduction of inflammation and skin fibrosis, halting the progression of pseudosyndactyly, was found in the treated mice. Based on these positive results, an investigator-initiated clinical trial on the therapeutic relevance of losartan in DEB was initiated (REFLECT trial, EUDRACT number 2015-003670-32). In addition, further safe and efficient molecules more directly targeting TGF-β activity are currently under pre-clinical testing, and clinical trials are to be expected soon.

Besides using small molecules, biologic products are also able to act as disease modifiers. Particularly potent agents in this context are mesenchymal stromal cells (MSCs), which have shown promise as disease-ameliorating drugs in EB because of their strong anti-inflammatory abilities ^28– 31^. Since these cells are also natural producers of collagen VII, given at a sufficient dose, they may also restore protein abundance at the dermo-epidermal junction zone ^29, 32– 35^. Interestingly, recent advances have identified a subpopulation of dermal-derived MSCs with more potent, tissue-regenerative abilities ^36, 37^. Clinical trials using these so-called ABCB5-positive MSCs in improving wound healing in DEB are expected to be initiated later this year.

Another EB subtype with a pathogenesis-driven search for novel therapies is EBS. The presence of keratin intermediate aggregates in keratinocytes of patients with EBS due to mutations in the keratin 5 and 14 genes were reported some years ago ^38– 40^. Similarly, patients suffering from plectinopathy-associated EBS with muscular dystrophy (EBS-MD) and mice lacking plectin in skeletal muscle display pathological desmin-positive protein aggregation. The group around Gerhard Wiche used the chemical chaperone 4-phenylbutyrate to treat muscle-specific, plectin-deficient mice and plectin-deficient myotubes, showing remarkable amelioration of the pathological phenotypes ^41^. 4-phenylbutyrate is an approved orphan drug, which is used to treat urea cycle disorders, as its metabolites offer an alternative pathway to allow the excretion of excess nitrogen. However, it has been tested not only in the urea cycle but also in other inherited disorders and was determined to be effective in progressive familial intrahepatic cholestasis ^42^; trials are ongoing for spinal muscular atrophy or thalassemia. The drug’s presumed mechanisms of action possibly include reduction of cellular stress and regulation of autophagy. Thus, the use of chaperones in EBS is currently being sought by several groups. In addition and possibly related to the pathogenic cellular aggregates, efforts are being made to target IL1β in EBS due to keratin mutations, since it has been shown to play a pathogenetic role in this EB subtype ^43^.

Although several clinical trials have now been initiated for individuals with EB, the treatment of the disease in most cases still remains symptomatic and based on preventive measures, together with symptomatic treatment of cutaneous and extracutaneous manifestations ^4^ and complications. A multidisciplinary team of experts from different European EB centers has prepared recommendations, grounded on available literature and expert opinion. They have been subsequently revised by a panel of external experts, using an online-modified Delphi method to generate consensus. The optimal management of patients is a prerequisite for them to benefit from the specific treatments that currently are under development ^7^. What has become apparent from the clinical studies conducted so far in EB is the lack of good data on natural history and disease severity biomarkers. As a substitute, excellent work on scoring the clinical severity of the disease has in some cases been used so far ^44– 47^.

## Abbreviations

DEB, dystrophic epidermolysis bullosa; EB, epidermolysis bullosa; EBS, epidermolysis bullosa simplex; JEB, junctional epidermolysis bullosa; MSCs, mesenchymal stromal cells; RDEB, recessive dystrophic epidermolysis bullosa; TGF, transforming growth factor
